# Short-term adaptation as a tool to improve bioethanol production using grass press-juice as fermentation medium

**DOI:** 10.1007/s00253-024-13224-0

**Published:** 2024-06-25

**Authors:** Ludovica Varriale, Doris Geib, Roland Ulber

**Affiliations:** Department of Mechanical and Process Engineering, Division of Bioprocess Engineering, Rhein-Palatinate Technical University Kaiserslautern-Landau, Gottlieb-Daimler-Str. 49, 67663 Kaiserslautern, Germany

**Keywords:** Lignocellulosic biomass, Bioethanol, *Saccharomyces cerevisiae*, Fermentation, Short-term adaptation

## Abstract

**Abstract:**

Grass raw materials collected from grasslands cover more than 30% of Europe’s agricultural area. They are considered very attractive for the production of different biochemicals and biofuels due to their high availability and renewability. In this study, a perennial ryegrass (*Lolium perenne*) was exploited for second-generation bioethanol production. Grass press–cake and grass press-juice were separated using mechanical pretreatment, and the obtained juice was used as a fermentation medium. In this work, *Saccharomyces cerevisiae* was utilized for bioethanol production using the grass press-juice as the sole fermentation medium. The yeast was able to release about 11 g/L of ethanol in 72 h, with a total production yield of 0.38 ± 0.2 g_Ethanol_/g_sugars_. It was assessed to improve the fermentation ability of *Saccharomyces cerevisiae* by using the short-term adaptation. For this purpose, the yeast was initially propagated in increasing the concentration of press-juice. Then, the yeast cells were re-cultivated in 100%(v/v) fresh juice to verify if it had improved the fermentation efficiency. The fructose conversion increased from 79 to 90%, and the ethanol titers reached 18 g/L resulting in a final yield of 0.50 ± 0.06 g_Ethanol_/g_sugars_ with a volumetric productivity of 0.44 ± 0.00 g/Lh. The overall results proved that short-term adaptation was successfully used to improve bioethanol production with *S. cerevisiae* using grass press-juice as fermentation medium.

**Key points:**

*• Mechanical pretreatment of grass raw materials*

*• Production of bioethanol using grass press-juice as fermentation medium*

*• Short-term adaptation as a tool to improve the bioethanol production*

**Supplementary Information:**

The online version contains supplementary material available at 10.1007/s00253-024-13224-0.

## Introduction

Nowadays, our society is dealing with important challenges such as environmental destruction, climate change, and insufficient fossil resources. In order to overcome these problems, the transition towards a bio-based economy has gained more attraction.

Lignocellulosic biomasses, also known as second-generation feedstocks (2G), are emerging due to the high availability, no-competition with the food/feed industry, and renewability. They are supplied from different resources such as agriculture and forestry residues, industrial and domestic wastes, and aquaculture (Cherubini et al. 2009).

In Europe, grassland and field grass cover one-third of the agriculture land in Europe (Kamm et al. 2016). Grass raw materials from herbaceous crops are very attractive due to the multiple growth cycles per year (Kongkeitkajorn et al. 2021), high biomass yield and productivity (Takara and Khanal 2015; Tsai et al. 2018), and low energy input (Ask et al. 2012). Further, the high content of structural polysaccharides, such as cellulose and hemicellulose, makes grass materials a potential substrate for energy and biochemical production (Prasertwasu et al. 2014; Tsai et al. 2018; Kongkeitkajorn et al. 2020). The general scheme to process green biomass is to separate the fiber-cake fraction and the juice fraction using mechanical pretreatments (Sieker et al. 2011). These pretreatment methods help to reduce the water content thus decreasing the costs for the transportations (Varriale et al. 2022). Moreover, mechanical pretreatments avoid the formation of inhibitory compounds which affect the microbial growth (Nielsen et al. 2015). For all the reasons mentioned above, perennial ryegrass can have an important role in the lignocellulose-to-ethanol process.

In 2017, petroleum resources provide 94% of total energy in the transportation sector, which accounts for 15% of the total pollutant emissions (International Energy Agency 2020; Sharma et al. 2020; Dobrescu et al. 2021). In this view, the Renewable Energy Directive Recast fixed a minimum target on the use of renewables to produce bioethanol of 14% by 2030 (European Parliament 2018; Dobrescu et al. 2021). According to this, it is expected that the ethanol bioproduction will surpass 130 billion l/year worldwide by 2030, with the United States and Brazil as main producers (OECD/FAO 2021; Tse et al. 2021). The baker’s yeast *Saccharomyces cerevisiae* is the conventional microorganism for bioethanol production. It has several appealing industrial qualities, such as fast growth, high production yields, and high tolerance to environmental stress factors. During the last years, several strategies have been used to improve its fermentative ability in lignocelluloses, such as rational engineering (mutagenesis) and evolutionary engineering (adaptive laboratory evolution (ALE) and the short-term adaptation) (Ask 2013). Evolutionary engineering is performed by applying a selective pressure for the desired phenotype in order to let the microorganism adapt to the condition(s) by natural evolution (Wang et al. 2023). If with the ALE the changes are incorporated into the genome, the short-term adaptation results in the selection of the phenotype(s) which is more adapted to the specific environmental factor (Nielsen et al. 2015; Nielsen 2016). The critical step in the short-term adaptation is the culture’s propagation which led to get cells with high fermentative efficiency and performance (Tomás-Pejó and Olsson 2015). Short-term adaptation has been used to obtain *S. cerevisiae* strains tolerant to inhibitors (Wallace-Salinas and Gorwa-Grauslund 2013; Gu et al. 2014; van Dijk et al. 2020; Almeida et al. 2023) or capable to co-ferment glucose and xylose (Klimacek et al. 2014; Nielsen et al. 2015; Dobrescu et al. 2021).

The aim of this work was to test the capability of *S. cerevisiae* to produce bioethanol using the press-juice from a perennial ryegrass (*Lolium perenne*) as fermentation medium. It was firstly examined the juice composition to verify the nutrient content. Then, it was investigated the possibility to improve the fermentation performance of *S. cerevisiae* adopting the short-term adaptation tool. For this purpose, the yeast was propagated in increasing concentration of press-juice. All the cultivations were evaluated in terms of sugars consumption, ethanol and by-product production, and biomass growth. Finally, physiological parameters were calculated and used to compare the fermentation performance of *S. cerevisiae* before, during, and after the adaptation.

## Materials and methods

### Raw material and juice preparation

The perennial ryegrass (*Lolium perenne*) was kindly provided by the Julius Kühn-Institute (Braunschweig, Germany). The production of the press-juice is described elsewhere (Varriale et al. 2022). The resulting juice was further processed before the use as fermentation medium. In particular, the juice was centrifuged twice at 4500 rpm for 20 min (Z38K, Hermle Labortechnik GmbH, Wehingen, Germany) to separate the slurry portion. To remove the remaining solid particles, the supernatant was filtered using filter paper (Macherey–Nagel, 185 mm). To prevent the loss of important nutrients, the sterilization process was carried out using a sterile filtration (Stericup®, 0.2 µm pore size, Merck Millipore, Massachusetts, USA). The obtained press- juice was stored at − 20 °C until further use.

### Microorganism and medium

The yeast *Saccharomyces cerevisiae* 3799 was obtained from the German Collection of Microorganisms and Cell cultures (DSMZ GmbH, Braunschweig, Germany). Preculture and control experiments were carried out in yeast peptone dextrose (YPD) medium with the following composition: yeast extract 10 g/L, peptone 20 g/L, dextrose 20 g/L (pH 6.2). Unless otherwise stated, all the chemicals were purchased from Carl Roth + Co KG (Karlsruhe, Germany), except dextrose which was purchase from Sigma-Aldrich (Merck KGaA, Darmstadt, Germany).

### Preculture and main culture preparation

Precultures were started from stock cryo-culture stored in 25% (v/v) glycerol at − 80 °C. They were grown aerobically in 100-mL baffled shake flasks for 24 h until the late-exponential phase was reached. The main cultures were inoculated to 0.1 OD (optical density) without pH adjustment. The main cultivations were performed anaerobically in 100-mL glass bottles with 50-mL working volume, tightly sealed with rubber septa. The bottles were sparged with pure nitrogen prior to inoculation. Both the precultures and the main cultures were incubated at 32 °C and 120 rpm (Ecotron, Infors AG, Bottmingen, Switzerland).

### Sampling and cell growth determination

Samples were regularly taken for the OD measurement. One milliliter of each sample was further centrifuged (14,000 rpm, room temperature, 7 min; Eppendorf, Hamburg, Germany), and the supernatant was stored at − 20 °C for HPLC (high-performance liquid chromatography) analysis. The cell dry weight (CDW) was determined in a separate experiment using YPD medium and aerobic condition for the generation of an OD-CDW correlation. The experiment was performed as follows: 5 mL of cell suspension was harvested by centrifugation (4500 rpm, 10 min, 4 °C, Z38K, Hermle Labortechnik GmbH, Wehingen, Germany). The cell pellet was washed with 5 mL and resuspended in 5-mL NaCl solution (9 g/L). Finally, the cell suspension was transferred to 15-mL dried tubes and then dried (both at 50 °C, 48 h) in the oven (Memmert GmbH, Schwabach, Germany) until a constant weight was reached. The CDW/OD_600_ correlation was determined to be 0.32 ± 0.02, and it was established in triplicates. The maximum specific growth rate (*µ*_MAX_) was determined in separate experiments in duplicates. Both the not adapted and adapted *S. cerevisiae* strains were cultivated in 100%(v/v) juice anaerobically. One milliliter sample was harvested and centrifuged (14,000 rpm, 7 min, room temperature, Eppendorf, Hamburg, Germany). The pellet was washed and resuspended in distilled water. The maximum specific growth rates were estimated from the slope of the linear regression between ln(OD) and time.

### Analysis of press-juice components and metabolites

The analysis of the press-juice was carried out with regard to pH, protein content, sugars, amino acids, cations, and anions. Before any analysis, the samples were filtered through a 0.2-µm pore size nylon filter (KX Syringe Filter Nylon, Cole-Parmer GmbH, Wertheim, Germany). The pH value was measured using a pH meter (Microprocessor pH 211, Hanna Instruments Deutschland GmbH, Vöhringen, Germany). The protein concentration was determined using the Bradford assay (Pierce® Coomassie Bradford Protein Assay kit, Thermo Scientific, Massachusetts, United States) and the bovine serum albumin as internal standard (Thermo Fisher Scientific, Massachusetts, United States). The absorbances of the calibration curve and of the samples were measured at 595 nm (Cary 60 UV–Vis, Agilent Technologies, USA). Sugar and product concentrations were analyzed by HPLC [Autosampler AS 6.1L (Knauer GmbH, Berlin, Germany), Azura pump P 6.1L (Knauer GmbH, Berlin, Germany)], and a refractive index detector (RI 101 Shodex, Kawasaki, Japan). The HPLC was equipped with an Aminex HPX-87H column (Bio-Rad, 300 × 7.8 mm, Hercules, California, USA) at 80 °C. The mobile phase was 2.5 mM H_2_SO_4_ at a flow rate of 0.6 mL/min. The instrument control and data evaluation were carried out with a Clarity software system (Data Apex, Prague, Czech Republic). Amino acids were separated using a resolve C18 column (150 × 3.9 mm, Waters Corporation, Milford, USA) with a SecurityGuard Cartridge (C18, 4 × 3.0 mm ID, Phenomenex, Torrance, California, USA) placed in a column heater CT 2.1 (Knauer GmbH, Berlin, Germany) set at 30 °C. The detection was performed using Azura photodiode-array detector DAD 2.1L at 230 nm (Knauer GmbH, Berlin, Germany). The analysis included a precolumn derivatization with ortho-phthaldialdehyde (OPA). The derivatization process was automated using the Azura AS 6.1L autosampler (Knauer GmbH, Berlin, Germany) and the own “mix method” option. The OPA reagent was prepared by weighting 270 mg OPA in a 50-mL volumetric flask. The reagent was dissolved in 5-mL ethanol. Then, 200-µL 2-mercapto-ethanol was added, and the final volume was filled up with 0.4 M borate buffer. The mobile phase consisted of solvent A (0.025 M sodium-acetate anhydrous and 0.025 M NaH_2_PO_4_ monohydrate) and solvent B (50% methanol). The pH value of solvent A was adjusted to 7 with 10 M NaOH, and then 21 mL of both tetrahydrofuran and methanol was added. The gradient elution program was as follows: from 0 to 50 min, solvent B changed linearly from 0 to 100%; from 50 to 55 min, solvent B was set as isocratic at 100%; from 55 to 60 min, solvent B changed linearly from 100 to 0%; from 60 to 67 min, solvent B was set as isocratic at 0%. The flow rate was 1.0 mL/min. To analyze the sample, it must be protein and particle-free. For this reason, the proteins were firstly precipitated by adding four parts of ice-cold methanol and one part of the sample. The sample was placed at − 20 °C overnight. Then, it was centrifuged for 10 min at 10,000 rpm (Eppendorf, Hamburg, Germany). Finally, it was diluted with 0.4 M borate buffer. A dilution of at least of 1:1 is necessary as the sample has to have a pH 10 for the derivatization. The derivatization was performed by adding 80 µL of the sample or the standard and 50 µL of OPA reagent. After 1 min, 40 µL was injected for the measurement. Cations and anions were analyzed by ion chromatography (IC) (930 Compact IC Flex, Metrohm GmbH & Co. KG, Germany) with an inline system for dialysis (930 Compact IC Flex, Metrohm, Filderstadt, Germany) and an IC Conductivity Detector (Metrohom, Filderstadt, Germany). Cations were measured with a cation column (Metrosep C6-250/4.0, Metrohm) using 4 mM HNO_3_ and 0.7 mM dipicolinic acid as mobile phase at a flow rate of 0.9 mL/min. Anions were measured with an anion column (Metrosep A Supp5-250/4.0, Metrohom) using 1 mM NaHCO_3_ and 3.2 mM Na_2_CO_3_ as mobile phase at a flow rate of 0.7 mL/min. The oven temperature in both cases was 35 °C. For each analysis, the samples were diluted to a concentration inside the external calibration range (using 2 mM HNO_3_ for cation determination).

### Propagation

**Fig. 1:**
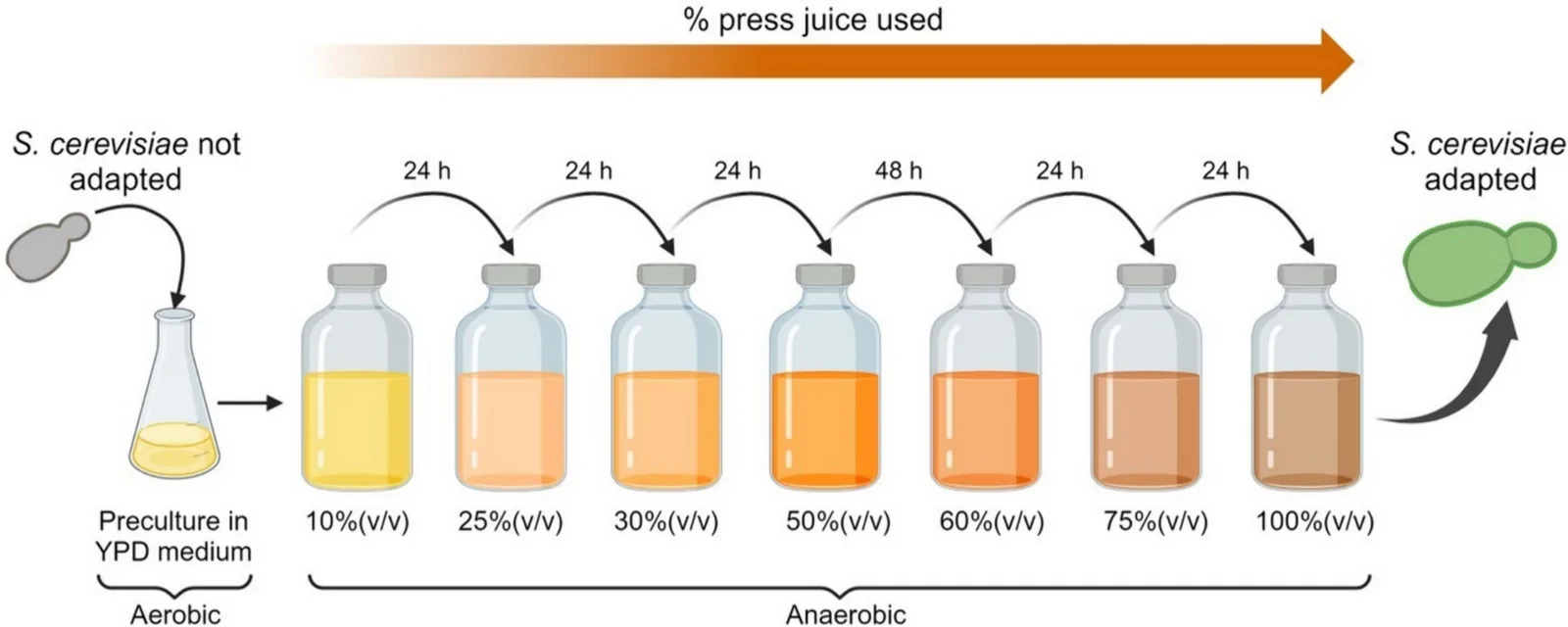
Propagation scheme of *S. cerevisiae* during short-term adaptation. The figure was generated using BioRender program

Propagation was performed in sequential anaerobic processes (Fig. 1). *S. cerevisiae* was cultivated in increasing concentration of grass press-juice (10%(v/v), 25%(v/v), 30%(v/v) 50%(v/v), 60%(v/v), 75%(v/v), 100%(v/v)). Ten percent (v/v) was used as starting point and not considered in bioethanol production. The cells were transferred to the next juice concentration after 24 h, except from the passage to 60%(v/v) which occurred after 48 h due to the lower yeast growth. After the cultivation in 100%(v/v), the adapted cells were harvested to prepare cryo-cultures. They were used to re-cultivate the cells in 100%(v/v) fresh juice to analyze the physiological parameters and fermentation capacity. The results were compared with those obtained from the not adapted *S. cerevisiae* strain. In every passage of the propagation, the juice was inoculated to 0.1 OD using the cells from the previous concentration.

### Data processing and evaluations

The percentage of sugars utilization (*S*_*u*_) was calculated according to Eq. (1)

1 $${S}_{u}=\frac{{S}_{0}-{S}_{t}}{{S}_{0}}\times 100$$

where *S*_0_ is the starting sugar concentration, and *S*_*t*_ is the sugar at the time.

Substrate consumption rates (*r*_*s*_) were calculated using the following equation:

2 $${r}_{s}=\frac{{S}_{t}-{S}_{0}}{t}$$

where *S*_*t*_ is the substrate concentration at specific time, and *S*_0_ is the substrate concentration at the beginning of the fermentation, and *t* is the time. Accordingly, the sugar consumption rate was characterized by a negative value as it described the decrease of sugar over time. The volumetric ethanol productivity was expressed in g/Lh (*Q*_EtOH_) and was estimated from the ratio between the ethanol concentration and the time at the end of the fermentations. The yields were calculated considering the g of product per g of total sugars consumed (glucose and fructose). Carbon balances included both biomass and metabolite yields, and it was assumed that 1 mol CO_2_ was formed per mol ethanol and acetate (Novy et al. 2017). For biomass yields, a C-molar weight of 26.4 g/Cmol was used (Lange and Heijnen 2001).

## Results

### Analysis of press-juice

**Fig. 2:**
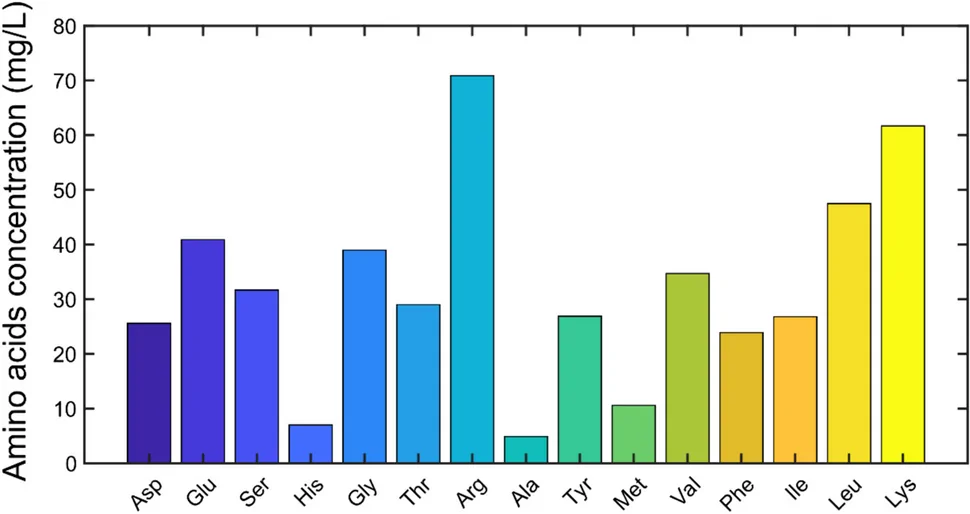
Amino-acid pattern in grass press-juice analyzed by HPLC

In order to use the press-juice as fermentation medium, it is important to know its composition in terms of sugars, proteins, amino acids, ions, and pH. The composition of the juice is reported in Table 1. The analysis of the sugars revealed that the juice was mainly composed by fructose (over 30 g/L) and small quantities of glucose (< 4 g/L). Moreover, since *S. cerevisiae* can metabolize only hexoses, no further sugars analysis was performed. With regard to the nitrogen content, both proteins and amino acids contributed to the total amount with 1008 mg/L and 480 mg/L, respectively. In particular, high concentrations of arginine, lysine, and leucine were found in the juice (Fig. 2). The analysis of the ions revealed that the elements present at higher concentrations were potassium (6.31 g/L), calcium (0.57 g/L), and magnesium (0.23 g/L) as cations and chloride (4.23 g/L), phosphate (2.75 g/L), and sulfate (1.42 g/L) as anions.

**Table 1:** Press-juice composition from the perennial ryegrass *Lolium perenne*. The pH value was 5.3

Sugars [g/L]	
Glucose	3.7
Fructose	36.4
Total	40.1
Protein [mg/L]	
Total	1008
Amino acids [mg/L]	
Total	479.2
Cation [g/L]	
Sodium	0.14
Ammonium	0.066
Potassium	6.31
Magnesium	0.23
Calcium	0.57
Total	7.32
Anion [g/L]	
Chloride	4.23
Nitrite	< 0.01
Nitrate	0.03
Phosphate	2.75
Sulfate	1.42
Total	8.43

### Time-course of bioethanol production using the press-juice as fermentation medium

**Fig. 3:**
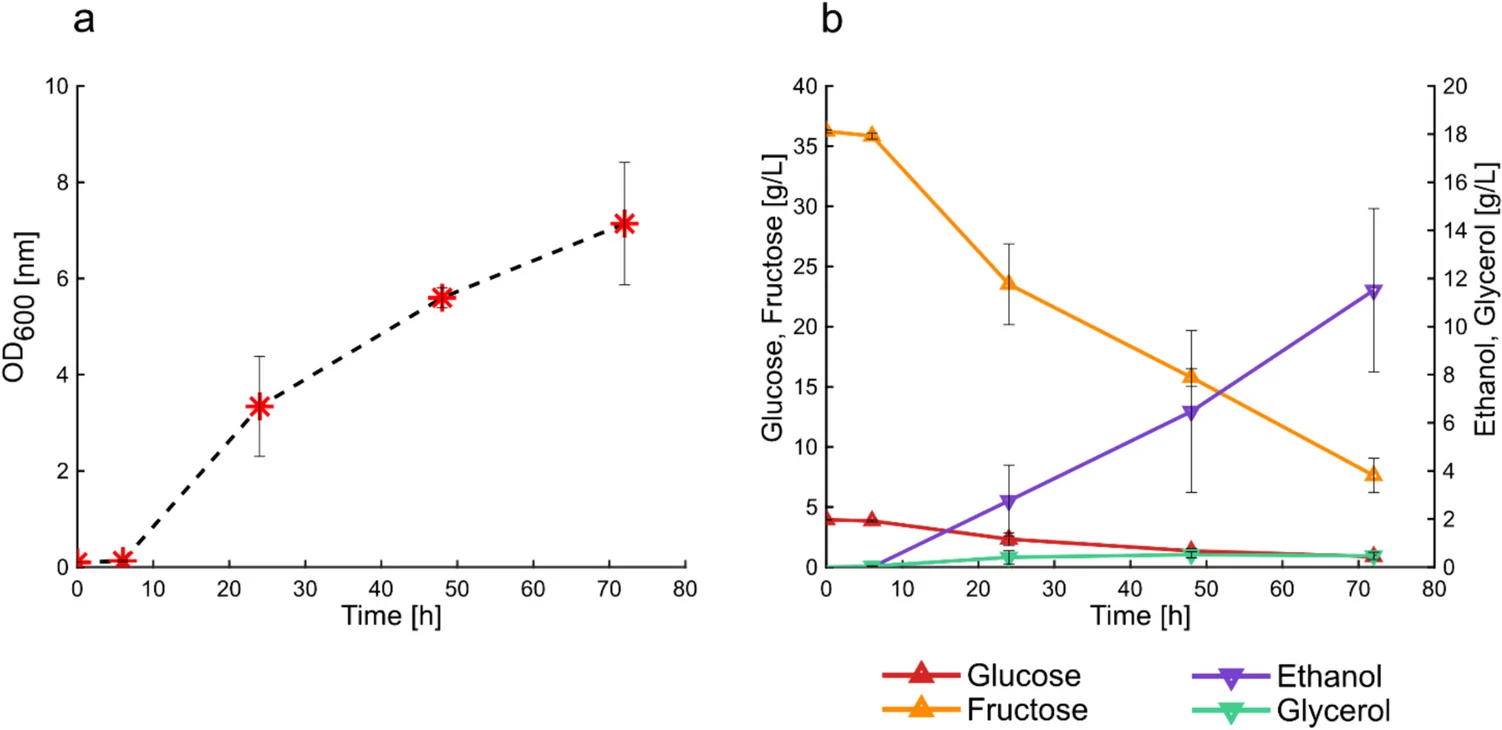
Cultivation of *S. cerevisiae* in 100% (v/v) press-juice. Growth curve (**a**), sugars consumption and products formation (**b**) in a 72-h fermentation. Data and error bars represent mean values and the standard deviations of triplicates. Growth conditions: 120 rpm, 32 °C, pH 5.3

To evaluate the potential of grass press-juice as fermentation medium, it was assessed whether *S. cerevisiae* was capable to grow and to produce bioethanol in 100%(v/v) press-juice. The cultivation of the yeast lasted for 72 h to ensure almost complete sugars consumption. The growth was monitored using the OD_600_ measurement. The growth profile showed that *S. cerevisiae* cultivated in press-juice as sole fermentation medium exhibited a lag-phase of about 6 h (Fig. 3a). This was further confirmed by the fact that no sugars were yet consumed (Fig. 3b). The lag-phase was followed by the exponential phase, which lasted for about 18 h. During the exponential-growth phase, glucose and fructose strongly decreased of about 41% and 35%, respectively, and the ethanol started to be produced. After 24 h of fermentation, *S. cerevisiae* continued to grow slower until the end of the cultivation. At this point, the glucose was completely depleted, 7.6 g/L fructose remained, and 11.5 ± 3.4 g/L of bioethanol was produced corresponding to a production yield of 0.38 g_Ethanol_/g_sugars_ (glucose and fructose).

### Growth profile during short-term adaptation

**Fig. 4:**
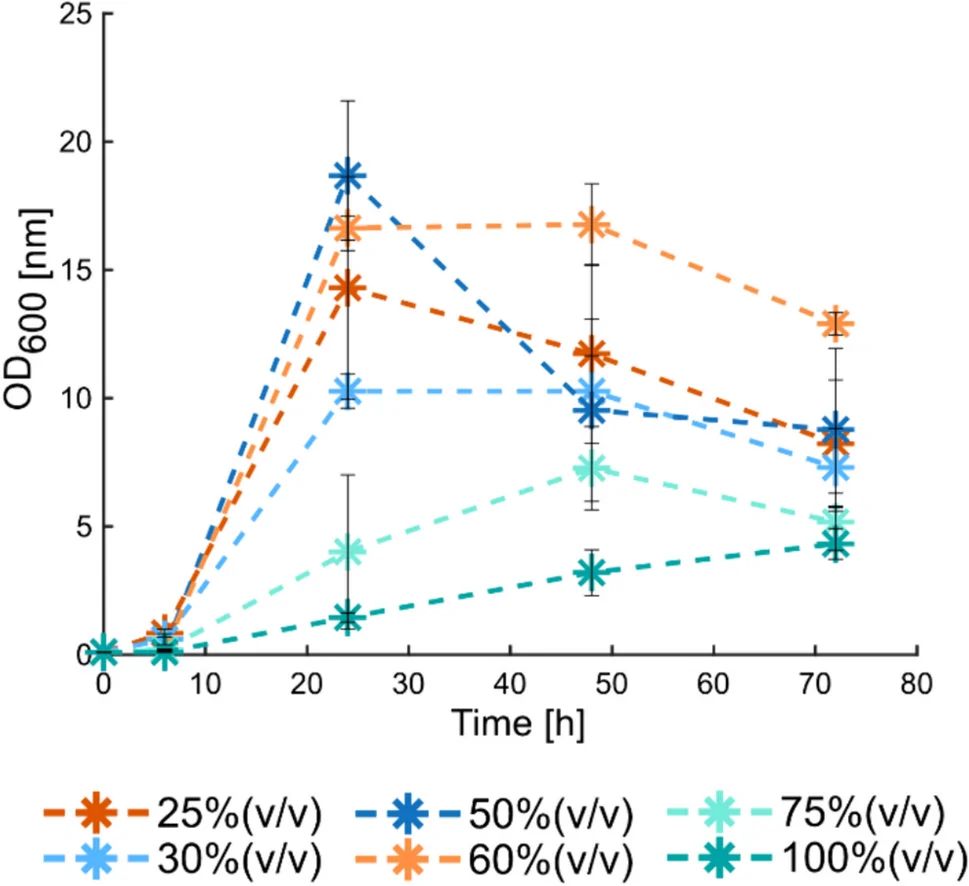
Growth profile of *S. cerevisiae* cultivated in different juice percentages during the propagation. Data and error bars represent mean values and the standard deviations of triplicates

Once it was proved that bioethanol could be produced using the press-juice as a fermentation medium, it was investigated the possibility of increasing the fermentation performance (in terms of sugars consumption and ethanol production) through short-term adaptation. For this purpose, the cells were propagated in increasing concentration of press-juice (25, 30, 50, 60, 75, and 100%(v/v)) in order to possibly obtain an adapted *S. cerevisiae* strain with improved fermentation capabilities. The growth profiles of *S. cerevisia*e did not change depending on the percentage of juice used (Fig. 4). In all cases the lag-phase lasted about 6 h, followed by the exponential phase. At 24 h, the highest cell density was recorded at 50%(v/v) of juice, followed by 60%(v/v), 25%(v/v), and 30%(v/v). Nonetheless, small differences could be observed in the beginning of the specific growth phase. In some juice percentages, the stationary phase started at 24 h and ended at 48 h, such as for 60 and 30%(v/v) press-juice. In other cases (25 and 50% (v/v)), the plateau was not distinct because of the few samplings, while at 75%(v/v) of press-juice, the exponential phase lasted until 48 h of cultivation. In all cases except at 100% (v/v), the cell densities started to decrease between 48 and 72 h of cultivation, thus meaning that the death phase took place. However, when the juice was used as sole fermentation medium, the exponential-growth phase lasted until 72 h.

### Sugars consumption and ethanol production during the short-term adaptation

**Fig. 5:**
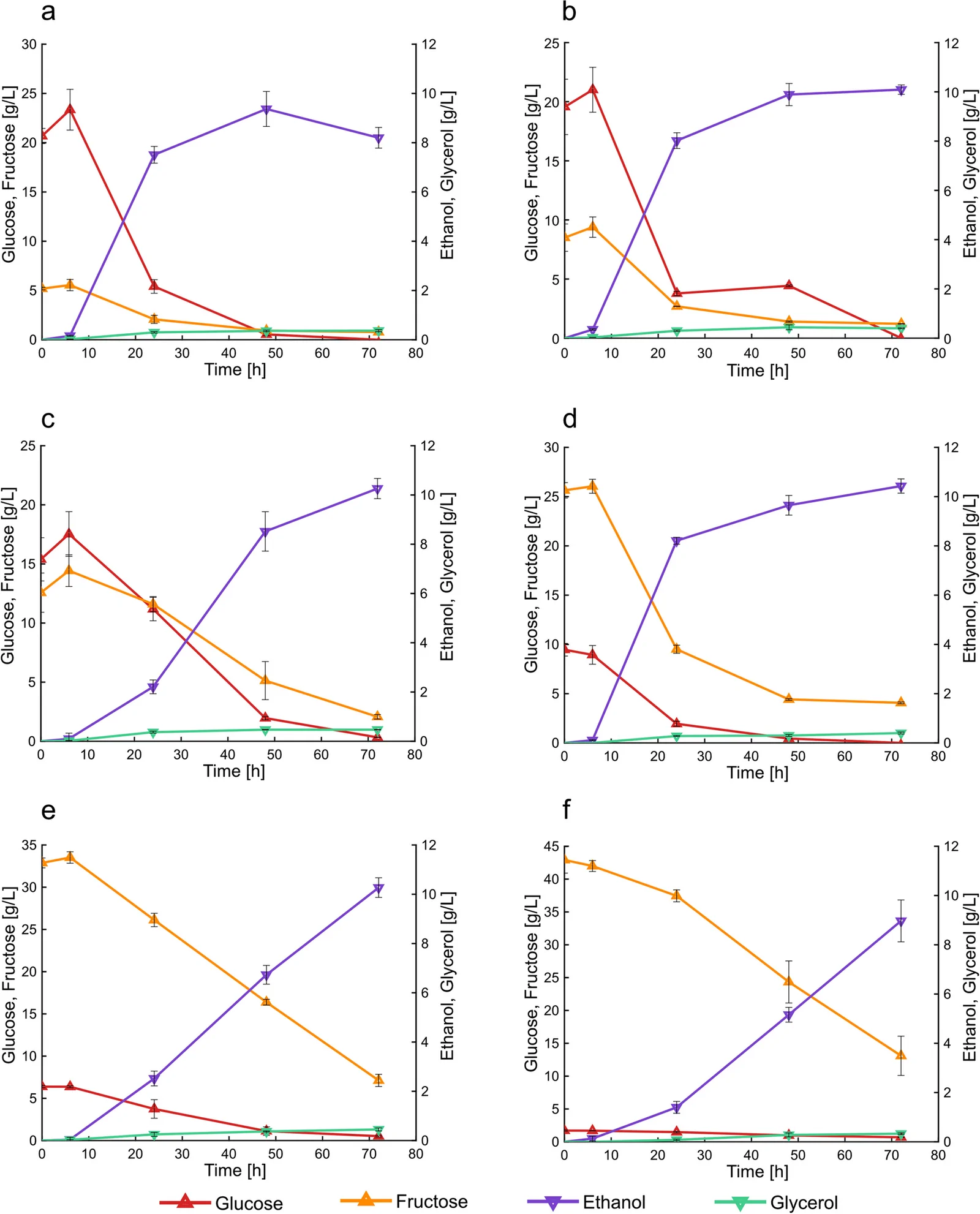
Cultivation of *S. cerevisiae* in different percentages of juice during short-term adaptation. Cultivation in 25%(v/v) press-juice (**a**), cultivation in 30%(v/v) press-juice (**b**), cultivation in 50%(v/v) press-juice (**c**), cultivation in 60%(v/v) press-juice (**d**), cultivation in 75%(v/v) press-juice (**e**), cultivation in 100%(v/v) press-juice (**f**). Data and error bars represent mean values and the standard deviations of triplicates. Growth conditions: 120 rpm, 32 °C, pH 5.9, 5.8, 5.6, 5.5, and 5.3 respectively

The time-course fermentations during the short-term adaptation strategy are illustrated in Fig. 5a–f. In all the percentages used for the propagation, glucose was almost totally consumed at the end of the fermentations. At 25%(v/v) and 30%(v/v) juice concentration, glucose was the predominant sugar in the medium and was depleted within the first 24 h (Fig. 5a, b). In the cultivation with 50%(v/v), glucose and fructose represented 56.8% and 43.2% of the total sugars, respectively. In this case, after 72 h, the glucose was almost totally consumed (0.3 g/L), and 2.01 g/L of fructose remained. The highest juice concentrations corresponded to higher fructose concentrations, and the sugar utilization was dependent on the percentage of juice used. In particular, between 25 and 60%(v/v) juice, the remaining fructose did not exceed 16% of the initial fructose concentration. A further increase in the juice percentage to 75%(v/v) and 100%(v/v) resulted in higher percentage of remaining fructose (21.6% and 30.5% respectively). The concentration of fructose remained at the end of the fermentation in each percentage is reported in Table 2. Regarding the products formation, ethanol was the primary product detected in the alcoholic fermentations (Fig. 5a–f). The titers and the yields for each juice percentages are reported in Table 2. In all the cultivations, its production started after 6 h according to the beginning of sugars consumption. Between 6 and 24 h, the solvent was rapidly produced. More than 78% of the total bioethanol was released within the first 24 h of fermentations at 25, 30, and 60% (v/v) of juice, corresponding to 7.51 ± 0.34 g/L, 8.02 ± 0.32 g/L, and 8.21 ± 0.14 g/L, respectively. Extending the time to 48 h resulted in the formation of 83.4%, 65.5%, and 57.5% of the total bioethanol when using 50, 75, and 100% (v/v) of juice concentrations, respectively. These results corresponded to 3.8-, 2.6-, and 3.6-fold product increase with respect to the first 24 h of fermentation. The ethanol production reached a plateau after 24 h of fermentation when 25–50%(v/v) were used. In the other conditions, it was still produced until the end of the fermentations due to the high sugars’ availability in the media. After 72 h, the solvent production increased by increasing the juice percentage, up to 75%(v/v). In terms of production yields, the highest values were recorded using 50%(v/v) of press-juice (0.41 g/g), followed by 30%, 60%, and 25% (v/v) of juice (Table 2). These results corresponded to 80% and 70% of the maximum theoretical ethanol yield, respectively. Aside from ethanol, glycerol and acetate are the main byproducts in the alcoholic fermentation. However, in this study, side-products (biomass, acetate and glycerol) were detected only in small amount.

**Table 2:** Physiological parameters of the fermentations with *S. cerevisiae* in different percentage of press-juice. *r*_*s*1_ and *r*_*s*2_ are the glucose and the fructose consumption rates, respectively. *C*_EtOH_ is the ethanol production at the end of the fermentations. *Q*_EtOH_ is the volumetric ethanol productivity. The yields were calculated considering the total sugar content

	25%(v/v)	30%(v/v)	50%(v/v)	60%(v/v)	75%(v/v)	100%(v/v)
Fructose remained (g/L)	0.78 ± 0.06	1.21 ± 0.02	2.07 ± 0.19	4.07 ± 0.09	7.12 ± 0.72	13.10 ± 2.99
*r*_*s*1_ (g/Lh)	− 042 ± 0.02^a^	− 0.40 ± 0.05^a^	− 0.28 ± 0.04^a^	− 0.22 ± 0.04^a^	− 0.11 ± 0.01^a^	− 0.02 ± 0.00^a^
*r*_*s*2_ (g/Lh)	− 0.09 ± 0.01^a^	− 0.15 ± 0.03^a^	− 0.16 ± 0.03^a^	− 0.44 ± 0.01^a^	− 0.42 ± 0.08^a^	− 0.39 ± 0.05^a^
*c*_EtOH_ (g/L)	8.20 ± 0.42	10.09 ± 0.19	10.26 ± 0.41	10.43 ± 0.29	10.27 ± 0.40	8.97 ± 0.85
*Q*_EtOH_ (g/Lh)	0.12 ± 0.02	0.14 ± 0.00	0.14 ± 0.01	0.14 ± 0.00	0.14 ± 0.01	0.12 ± 0.00
*Y*_EtOH_ (g/g)	0.36 ± 0.06	0.38 ± 0.04	0.41 ± 0.06	0.36 ± 0.05	0.22 ± 0.01	0.29 ± 0.02
*Y*_Glycerol_ (g/g)	0.014 ± 0.003	0.014 ± 0.000	0.018 ± 0.002	0.014 ± 0.000	0.015 ± 0.001	0.011 ± 0.000
*Y*_AceticAcid_ (g/g)	n.d	n.d	n.d	n.d	n.d	n.d
Y_Biomass_ (g/g)	0.20 ± 0.06^a^	0.13 ± 0.03^a^	0.15 ± 0.07^a^	0.19 ± 0.04^a^	0.09 ± 0.01^a^	0.05 ± 0.01^a^

*n.d.* not detectable

^a^Parameters were determined at 48 h

### Sugars consumption and ethanol production by the adapted strain of *S. cerevisiae*

**Fig. 6:**
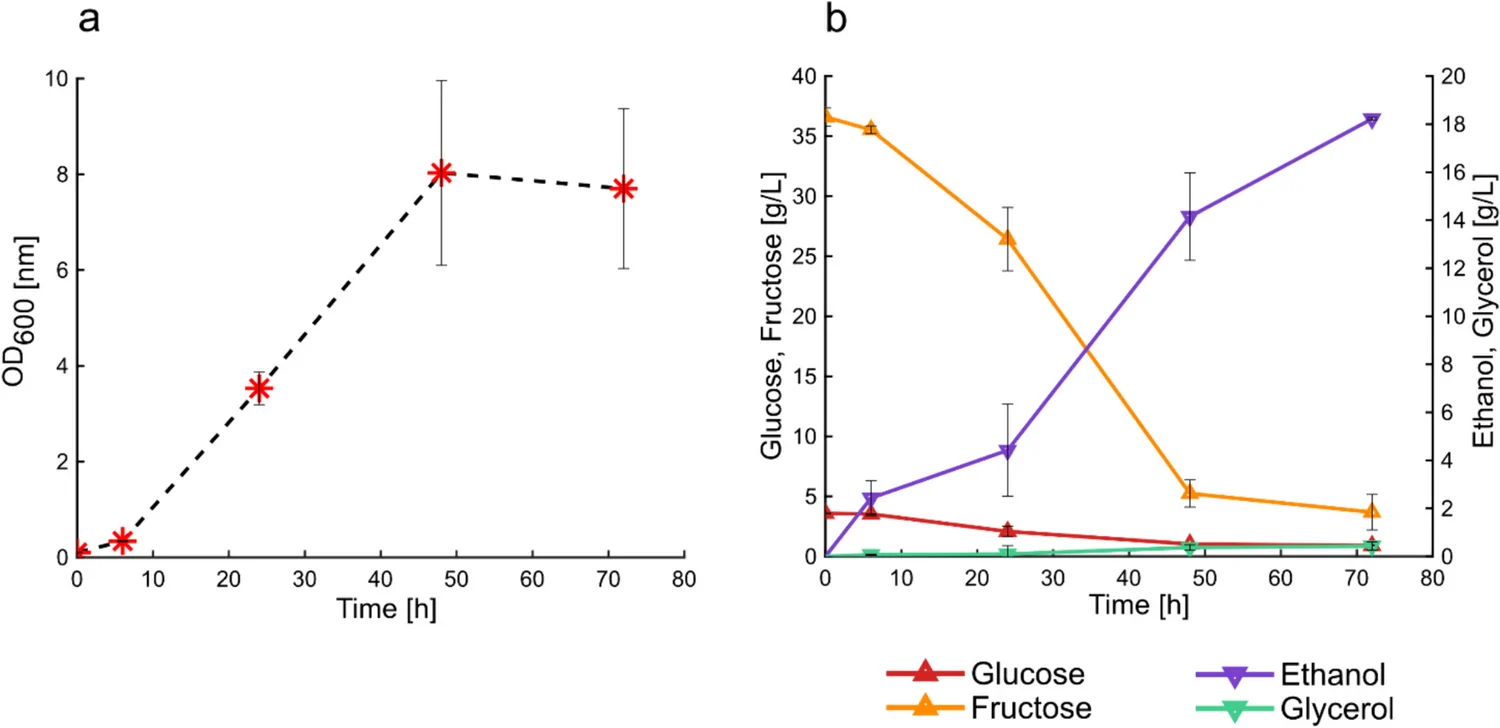
Cultivation of adapted *S. cerevisiae* in 100%(v/v) press-juice. Growth curve (**a**), sugars consumption and products formation (**b**) during 72-h fermentation. Data and error bars represent mean values and the standard deviations of triplicates. Growth conditions: 120 rpm, 32 °C, pH 5.3

After the adaptation, the cells cultivated in the last step of propagation were recovered and re-cultivated in fresh 100%(v/v) press-juice. The experiment was performed to assess if the yeast adapted to the set environmental conditions. According to this, cell density, sugars utilization, and product formation were evaluated (Fig. 6a, b).

**Fig. 7:**
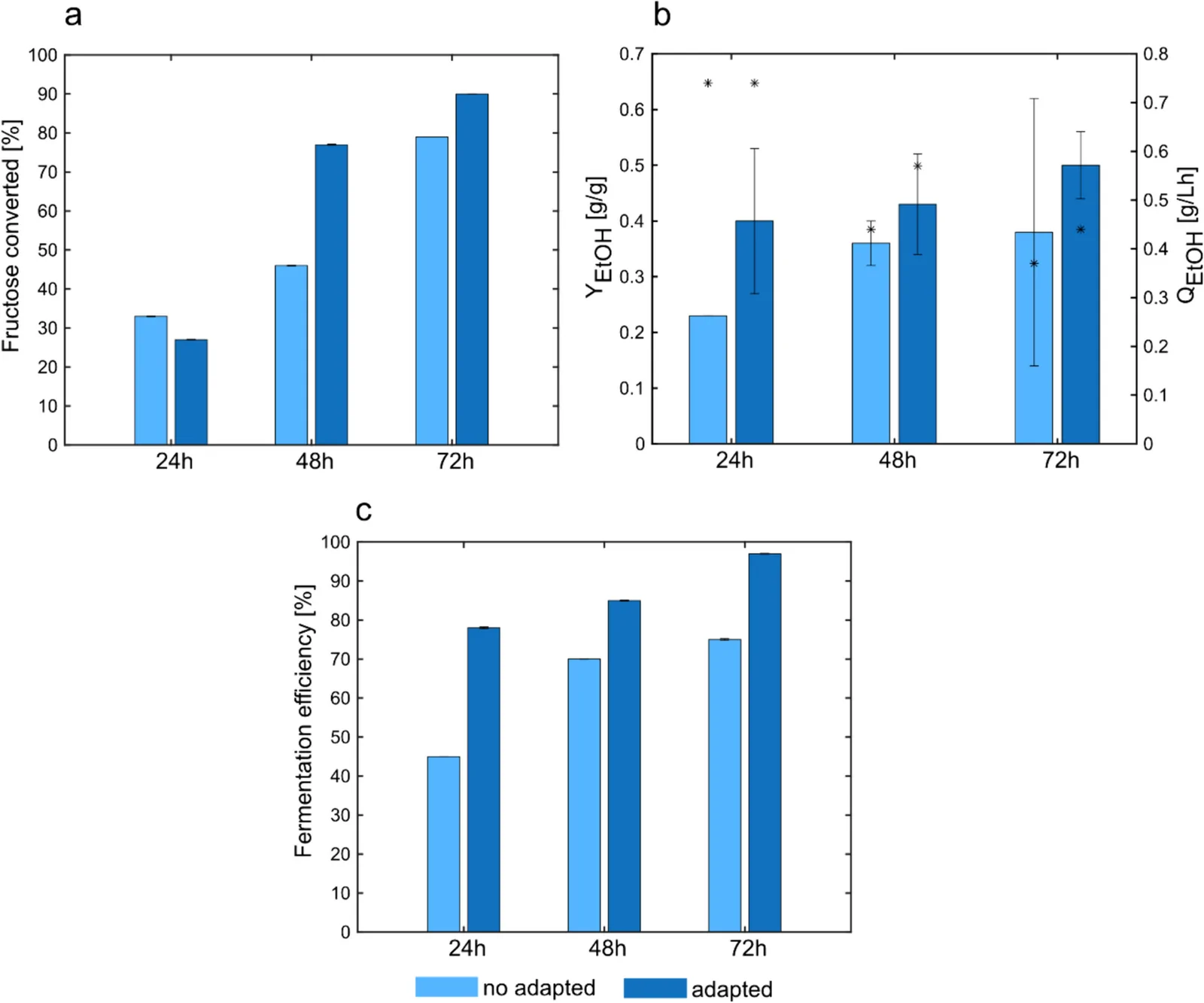
Percentages of fructose consumed with respect to the initial concentration (**a**), ethanol yields (bars) and productivities (stars) (**b**), fermentation efficiencies at 24 h, 48 h, and 72 h of cultivation (**c**). The percentage of fructose consumed was calculated as mentioned in Eq. 2

In contrast to the not adapted *S. cerevisiae*, the growth of the adapted *S. cerevisiae* started immediately (Fig. 6a). This was also supported by the initial utilization of sugars, especially fructose. In the first 24 h of cultivation, approximately 13 g/L of fructose were metabolized. The fructose consumption led to a bioethanol production of 4.42 ± 1.92 g/L. However, the exponential-growth phase lasted until 48 h, resulting in further fructose consumption (about 20 g/L) and increased solvent production (14.15 ± 1.82 g/L). Although notable differences between the not adapted and the adapted strains were observed in the case of ethanol production, the titers and the yields for glycerol and biomass were almost identical (Table 3). Figure 7 a shows the percentages of fructose conversion in the not adapted and adapted *S. cerevisiae* strains. At the end of the cultivations, the not adapted strain consumed about 79% of the total fructose and the adapted strain converted about 90%. The faster and broadly fructose consumption resulted in higher product titer (Table 3), as well as higher product yields and productivities (Fig. 7b), reaching almost the maximum theoretical ethanol yield of 0.51 g_Ethanol_/gs_ugar(s)_ (Koppram et al. 2013). At 24 h, the bioethanol productivities between the two strains did not differ considerably, reaching about 0.74 g/Lh. However, in both cases, the productivities decreased overtime, and the adapted strain showed higher values for all the time-points considered (Fig. 7b). To better compare the fermentation ability of both strains, the fermentation efficiency was calculated by dividing the bioethanol yield for the theoretical yield. The results are shown in Fig. 7c. In particular, at 24 h, the fermentation efficiencies were 45% for the not adapted strain and 78% for the adapted strain. The fermentation efficiencies were higher in the adapted strain than in the not adapted at the other time points. In particular, for the adapted strain, the values reached 85% at 48 h and 97% at 72 h.

**Table 3:** Physiological parameters of the not adapted and the adapted *S. cerevisiae* strain. *r*_*s*1_ and *r*_*s*2_ are the glucose and the fructose consumption rates, respectively. *C*_EtOH_ is the ethanol production at the end of the fermentations. *Q*_EtOH_ is the volumetric ethanol productivity. The yields are calculated considering the total sugar content

	*S. cerevisiae* not adapted	*S. cerevisiae* adapted
Fructose remained (g/L)	7.62 ± 1.43	3.68 ± 1.48
*µ*_MAX_ (h^−1^)	0.30 ± 0.00	0.26 ± 0.03
*r*_*s*1_ (g/Lh)^a^	− 0.05 ± 0.00	− 0.05 ± 0.00
*r*_*s*2_ (g/Lh)^a^	− 0.36 ± 0.06	− 0.58 ± 0.1
*c*_EtOH_ (g/L)	11.5 ± 3.4	18.2 ± 0.06
*Q*_EtOH_ (g/Lh)	0.37 ± 0.04	0.44 ± 0.00
*Y*_EtOH_ (g/g)	0.38 ± 0.2	0.50 ± 0.06
*Y*_Glycerol_ (g/g)	0.014 ± 0.00	0.013 ± 0.00
*Y*_AceticAcid_ (g/g)	n.d	n.d
*Y*_Biomass_ (g/g)^a^	0.09 ± 0.01	0.08 ± 0.01
C-recovery (%)^a^	72%	95%

*n.d.* not detectable

^a^Parameters were determined at 48 h

## Discussion

The grass raw material was mechanically pretreated to separate the solid fraction (press-cake) and the liquid fraction (press-juice). Mechanical pretreatment was chosen to avoid the formation of inhibitory compounds that can reduce the growth capacity of the yeast in lignocellulosic-derived media. In a biorefinery concept, the integrated utilization of both the solid and the liquid fractions would be beneficial. Several examples of the utilization of the press-cake fraction can be mentioned such as for animal feed (Damborg et al. 2020), for biogas production (Damborg et al. 2020), and as substrate in enzymatic hydrolysis and in solid-state fermentation steps (Kongkeitkajorn et al. 2020; Taufikurahman et al. 2020). In this work, the utilization of the press-juice as medium in fermentation processes was investigated. Firstly, the nutritional potential of the juice was evaluated. For this reason, the analyses of sugars, proteins, amino acids, and ions were carried out.

The higher concentration of fructose compared to glucose was found also by Sieker et al. Volkmar et al. Putra et al. and Sipos et al. (Sipos et al. 2009; Sieker et al. 2011; Putra et al. 2017; Volkmar et al. 2023). The nitrogen source was provided by organic nitrogen compounds, i.e., proteins and amino acids, and both are required for yeast metabolism and growth (Takagi 2019; Rojas et al. 2021). In particular, amino acids are essential components in liquid fermentation processes, and they are usually supplied by yeast extract in high amount (Tomé 2021). However, yeast extract is expensive, and its addition to standard fermentation media increases the overall process costs. Among the amino acids, arginine and leucine were quantified at higher amounts. Arginine is known to work as a cryoprotector which acts by suppressing the protein aggregation (Takagi 2019). Leucine belongs to the branched-chain amino acids family. It plays an important role as building block for protein synthesis and in the metabolism of fatty acids (Zhang et al. 2017; Takagi 2019). Ions are also essential for cellular metabolism as they act as cofactors for many enzymes (Cao and Liu 2013). Similar trends were found out by Boakye-Boaten et al. (2016). The authors reported a concentration of 1968.2 ppm of calcium, 93,083.3 ppm of potassium, 19,658.3 of magnesium (corresponding to 1.97 g/L, 93.08 g/L, and 19.66 g/L, respectively) (Boakye-Boaten et al. 2016). On the contrary, Cao and Liu (2013) found out in sweet sorghum juice lower concentration of calcium (0.35 g/L) and potassium (3.23 g/L) (Cao and Liu 2013). On the other hand, the authors got higher concentration of other elements such as sodium (1.01 g/L), while almost the same concentration of potassium was detected (6.16 g/L) (Cao and Liu 2013). Discrepancies in grass juice composition can be attributed to the different grass species, location, climate, and maturation stage (Prasertwasu et al. 2014; Takara and Khanal 2015). According to these findings, it is possible to state the potential of the grass press-juice as fermentation medium, since it contains many components usually present in the standard cultivation media.

The growth of *S. cerevisiae* in the pressed juice was monitored by measuring the OD. The resulting growth curve revealed that the yeast had a longer lag-phase compared to the growth in standard complex medium (Fig. S1). This could be justified by the fact that the preculture was grown in YPD medium, so the yeast would need more time to adapt to the juice. During the propagation of *S. cerevisiae* in increasing concentration of press-juice, the growth profile was monitored. It was possible to state that the lower the juice concentration, the higher was the cell density. These results were probably associated to a better nutrients balance between the components in the press-juice and those in the YPD medium. These results are consistent with the study of Tan et al. who found that maximum bioethanol production was reached at 80%(v/v) in comparison to 100%(v/v) banana juice (Tan et al. 2019). The authors suggested that this was likely due to the presence of heavy metals which cause a detrimental effect on the yeast growth (Tan et al. 2019). However, a study of Boakye-Boaten et al. showed that at 90%(v/v) Miscanthus juice, both the cell concentration and growth rate were higher compared to the lower juice percentage of 50%(v/v) (Boakye-Boaten et al. 2016). The authors supposed that this was due to the higher availability of minerals and other compounds which stimulate the growth (Boakye-Boaten et al. 2016). Discrepancies with these results may be related to the type of grass, yeast strain used, and growth conditions. Nevertheless, in our study, the cells were continuously propagated, meaning that the cells from the previous juice percentage were used as preculture for the next one. It is highly possible that not all the cells passed were viable; therefore, further experiments regarding cell viability should have been performed. For instance, Nielsen et al. carried out experiments on cell quantification and viability using cell counting and methylene blue staining, respectively (Nielsen et al. 2015). The authors found out that the number of cells decrease with the increase of hydrolysate liquor percentage. However, when the authors assessed the viability, they found out that number of viable cells increased by increasing the amount of hydrolysate liquor. These results were explained by assuming that these cells might be more tolerant to those conditions (Nielsen et al. 2015).

During the propagation, sugars consumption and ethanol production were evaluated too. The sugar consumption rates were calculated according to Eq. 2 and resulted in negative values. The negative values expressed the decrease of sugar concentration in the fermentation environment due to substrate metabolization by the yeast cells. In all the percentages, the residual glucose was around 8% of the initial concentration, except when 100%(v/v) juice was used (40% remained). This could be attributed to the low initial glucose concentration (1.7 g/L) in the press-juice, which may be insufficient for its metabolization (Meijer et al. 1998). At 50% (v/v) press-juice, the concentration of glucose and fructose was nearly the same; however, the residual fructose was 16.4% with respect to the initial concentration. Nonetheless, with increasing juice percentage, more fructose remained at the end of fermentations. This result confirms that glucose is the preferred substrate, although *S. cerevisiae* is able to consume both glucose and fructose. Similar results were reported by Putra et al. who cultivated *S. cerevisiae* in a 1-L reactor using date fruit syrup as fermentation medium containing 142.5 g/L total sugars (glucose and fructose). The authors pointed out that 83.5% of the fructose remained after 96 h of fermentation (Putra et al. 2017). The amount of fructose not metabolized can be explained by the fact that during the last phase of the fermentations, the yeast is overstressed due to nitrogen starvation and/or to the increased ethanol concentration (Berthels et al. 2008; Tronchoni et al. 2009). These conditions, combined with the high fructose-to-glucose ratio, may lead to stuck fermentations (Berthels et al. 2004). Another reason could be related to the diverse sugar internalization. Guillaume et al. found out that glucose and fructose have different consumption trends due to differences in the transportation across the membrane (Guillaume et al. 2007). Berthels et al. asserted that the dissimilarity was associated to differences in the hexose phosphorylation (Berthels et al. 2008).

The consumption of the sugars is related to the bioethanol production, which is the primary product of yeast fermentation. During the propagation, the bioethanol production started after the end of the lag-phase (6 h). However, when *S. cerevisiae* was cultivated in YPD medium, the ethanol production started after 4 h (Fig. S2). Therefore, when press-juice was used as fermentation medium, the yeast required a longer phase to adapt its metabolism. The highest yield value of 0.41 g_Ethanol/_g_sugars_ was obtained when 50%(v/v) was used, which corresponded to ethanol production of 10.26 ± 0.41 g/L. This ethanol yield corresponded to 80% of the maximum theoretical ethanol yield. Gomez-Florez et al. achieved a yield of 0.27 g_Ethanol_/g_Carbohydrates_ using juice sugar corn supplemented with 3 g/L yeast extract (Gomez-Flores et al. 2018). Tan et al. reached a yield of 0.33 g_Ethanol_/g_sugar_ using banana frond juice in a 2-L bioreactor (Tan et al. 2019). Bautista et al. utilized corn stalk juice and immobilized *S. cerevisiae* to produce ethanol, reaching 0.45 g_Ethanol_/g_sugar_ (Bautista et al. 2022). These findings demonstrate that all the percentages used for propagation were optimal for ethanol production.

Once propagated, the yeast cells were re-cultivated in 100%(v/v) to assess if they acquired enhanced fermentation capabilities. So far, short-term adaptation has been used as a strategy to develop a strain with novel characteristics which reflect its adaptation to a specific condition. However, in this study, the potential of short-term adaptation as a tool to improve the fermentation performance of *S. cerevisiae* in grass press-juice was explored. The sugars consumption and the bioethanol production before and after the adaptation were compared to evaluate the yeast’s performance. The improvements in the adapted strain were remarkable, resulting in a higher ethanol titer and a higher ethanol yield (0.50 g_Ethanol_/g_sugars_). So far, reaching a production yield close to the theoretical one in unconventional media has not been obvious. The overall results indicate that short-term adaptation can be exploited to enhance fermentation processes.

## Supplementary Information

Below is the link to the electronic supplementary material.

Supplementary file1 (PDF 129 KB)

## Data Availability

The data generated and analyzed in this study are available from the corresponding author upon request.
