# Supplementary material for: Short-term adaptation as a tool to improve bioethanol production using grass press-juice as fermentation medium
Source: Appl Microbiol Biotechnol. 2024 Jun 25;108(1):393. doi: 10.1007/s00253-024-13224-0 (PMC11199226; doi:10.1007/s00253-024-13224-0)
Supplement: Supplementary file 1 — Supplementary file1 (PDF 129 KB) [file 253_2024_13224_MOESM1_ESM.pdf]

Applied Microbiology and Biotechnology

Short-term adaptation as a tool to improve bioethanol production using grass press-juice as fermentation medium

Ludovica Varriale, Doris Geib, Roland Ulber*

Rhein-Palatinate Technical University Kaiserslautern-Landau; Department of Mechanical and Process Engineering, Division of Bioprocess Engineering, Gottlieb-Daimler-Str. 49, 67663 Kaiserslautern

*corresponding author

ulber@mv.uni-kl.de

Tel.: +49 631 205-4043/-4044

Fax: +49 631 205-4312

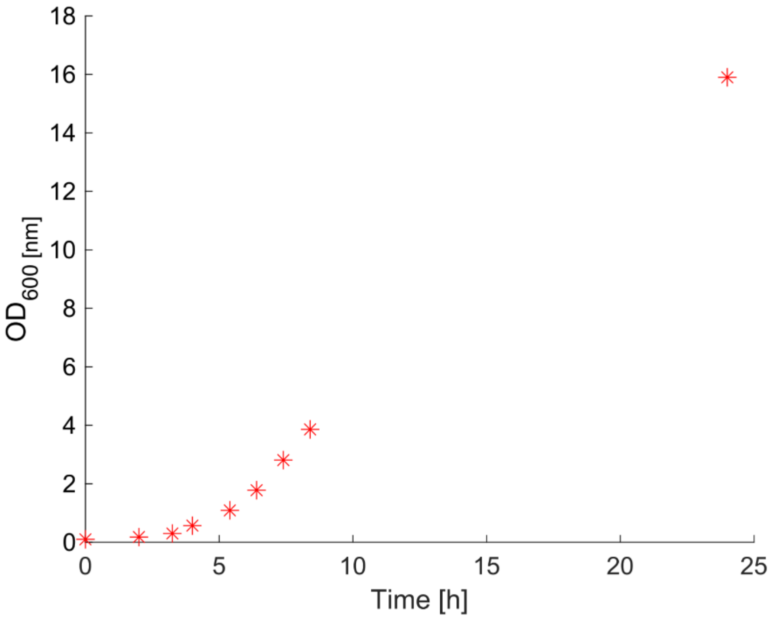

Fig. S1 Growth curve of *Saccharomyces cerevisiae* in YPD medium. Growth conditions: 120 rpm, 32°C, pH 6.2

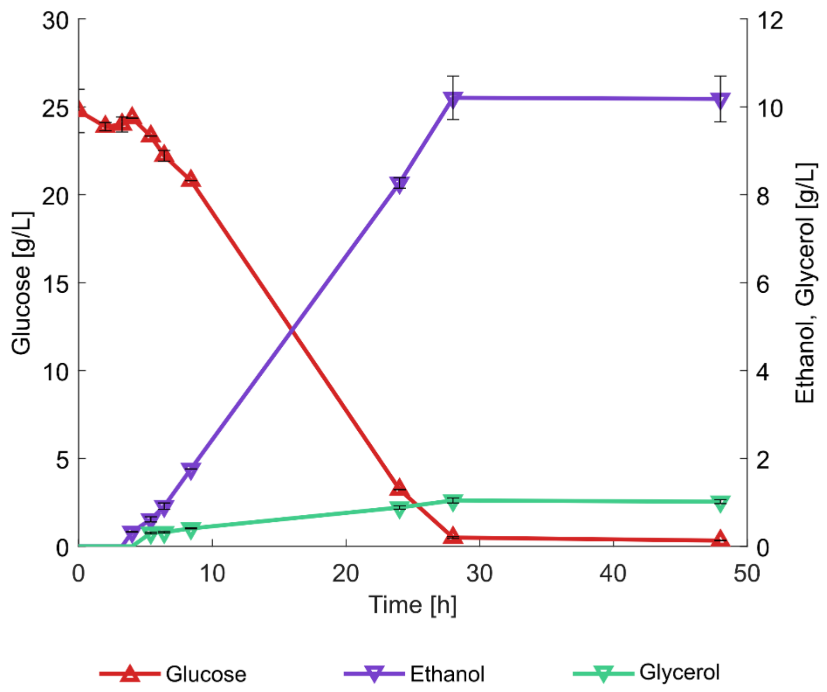

Fig. S2 Glucose consumption and bioethanol production in YPD medium. Growth conditions: 120 rpm, 32°C, pH 6.2
